# Magnetron Sputtered CsPbCl_3_ Perovskite Detectors as Real-Time Dosimeters for Clinical Radiotherapy

**DOI:** 10.1016/j.zemedi.2022.02.003

**Published:** 2022-03-31

**Authors:** Mara Bruzzi, Nicola Calisi, Matteo Latino, Naomi Falsini, Anna Vinattieri, Cinzia Talamonti

**Affiliations:** a Dipartimento di Fisica e Astronomia, Università degli Studi di Firenze, Via G. Sansone 1, 50019, Sesto Fiorentino, FI, Italy; b I.N.F.N. Sezione di Firenze Via G. Sansone 1, 50019, Sesto Fiorentino, FI, Italy; c Dipartimento di Ingegneria Industriale, Università degli Studi di Firenze, Via S. Marta 3, 50139, Firenze, Italy; d Dipartimento di Scienze dell’Informazione, Università degli Studi di Firenze, Via S. Marta 3, 50139, Firenze, Italy; e ENEA, Italian National Agency for New Technologies, Energy and Sustainable Economic Development, Fusion and Technology for Nuclear Safety and Security Department, Nuclear Safety, Security and Sustainability Division, via Martiri di Monte Sole 4, 40129, Bologna, Italy; f Dipartimento di Scienze Biomediche Sperimentali e Cliniche “Mario Serio”, Università degli Studi di Firenze, Firenze, Italy

**Keywords:** Radiation detectors, Dosimeters, Radiotherapy, Metal halide perovskite, Thin films

## Abstract

The aim of this study is to investigate the feasibility of manufacturing thin real-time relative dosimeters for clinical radiotherapy (RT) with potential applications for transmission monitoring *in vivo* dosimetry and pre-treatment dose verifications. Thin (≈1 μm) layers of a high sensitivity, wide bandgap semiconductor, the inorganic perovskite CsPbCl_3_, have been grown for the first time by magnetron sputtering on plastic substrates equipped with electrode arrays. Prototype devices have been tested in real-time configuration to evaluate the dose delivered by a 6 MV photon beam from a linear accelerator. Linearity of the charge with the dose has been verified over three order of magnitudes, linearity of the current signal with the dose rate has been also successfully tested in the range 0.5-4.3 Gy/min. The combination of high sensitivity per unit volume and wide bandgap provides high signal-to-noise ratios, up to 70, even at moderate applied voltages. The Schottky diode configuration allows the detector to operate without bias voltage (null bias).The blocking-barrier structure allows to confine the active volume within sub-millimetric sizes, a quite attractive feature in view to increase granularity and achieve the high spatial resolutions required in modern RT techniques. All the above-mentioned features indeed pave the way to a novel generation of flexible, transmission, real time dosimeters for clinical radiotherapy.

## 1 Introduction

Accurate radiation monitoring systems are required in radiotherapy (RT), in view to get a precise knowledge of the radiation beams used to treat patients and to verify the actual dose delivered both pre-treatment and in-vivo dosimetry [1], [2]. Recent advances in technology and research are directed towards delivering ever more tailored radiation treatments in an ever easier and faster way, to improve the RT efficacy [3]. Typically, either 6–24 MeV electrons or 6–25 MV Bremsstrahlung photon beams are delivered by linear accelerators (linac) in a pulsed structure with frequencies opportunely selected to get dose-rates, *D*_*r*_, in the range 0.5–4 Gy/min, nonetheless novel accelerators may reach dose rates up to ≈ 20 Gy/min, in view to reduce uncertainties due to patient motion and improve workflow efficiency. Moreover, novel applications with ultrahigh dose-rate (FLASH) radiotherapy have been taken recently into consideration [4]. The principle of operation of high-energy real-time monitoring dosimeters is to collect a charge accelerated by an electric field applied between two electrodes when a gas/semiconductor volume is subject to a flux of high energy particles [5]. At present, state-of-art dosimeters able to measure absorbed dose in real-time are mainly ionization chambers as well as silicon and diamond diodes [7], [6], [8]. During irradiation, the current signal *I* is read-out at constant voltage applied. For a proper operation, the current signal must be linearly dependent on dose – rates and charge *Q* obtained by integrating the current on the delivery time must be linearly dependent on the dose *D*. The sensitivity of the device is defined as the slope of the curve: S=QD=IDr. Modern techniques as Intensity Modulated RadioTherapy (IMRT) and Volumetric Modulated Arc Therapy (VMAT) involve intensity modulated beams and rotational treatments, characterized by high dose gradients, strong variations in space and time of both dose-rate and beam energy spectrum [1], [3]. Moreover, volumetric measurements require flexible devices adapting to the 3D patient/phantom geometry, while *in-vivo* needs thinned devices to allow for transmission measurements. Here, flexible stands for bendable, namely the device, being on plastic sheets, is not forced to cover flat areas but may also adaptable to curved geometries. This has the advantage to widen its range towards complex 3D configurations. To increase accuracy in regions of high dose gradients and in transmission, dosimetric arrays should minimize both active surfaces and thickness without significantly reducing the sensitivity *S*. This is obtained by selecting materials with a high sensitivity per unit volume. To increase accuracy at low dose-rates, the noise contribution to the signal, due to the dark current, should also be minimized. This can be obtained using wide-bandgap semiconductors (E_g_ ≥ 3 eV), as e.g. SiC and diamond, materials usually characterized by a significantly lower sensitivity per unit volume than silicon [6], [8]. Finally, in terms of flexible devices, research advances recently promoted the investigation of novel materials directly deposited on substrates equipped with electrode arrays [9], [10], [11] and with active volumes with sub-millimetric size [12], in views to increase granularity and achieve the high spatial resolutions required in modern RT techniques .

The aim of this study is to investigate feasibility of manufacturing thin real-time dosimeters for clinical radiotherapy (RT) applications as transmission monitoring *in-vivo* dosimetry and pre-treatment dose verifications. Perovskite materials, with AMX_3_ structure, A = Cs^+^, CH_3_NH_3_^+^ methylammonium, CH(NH_2_)_2_^+^, M metal cations (Pb_2_ + or Sn_2_ + ), and X halide anions (I^−^ or Br^−^ or Cl), besides their excellent optoelectronic properties [13], are characterized by easy deposition methods, allowing to deposit on almost any kind of substrates while properly tailoring size and thickness [14]. This paper shows results with cesium lead chloride (CsPbCl_3_), a wide gap inorganic perovskite semiconductor able to provide both a high sensitivity per unit volume and low leakage currents at room temperature. Prototypes manufactured in this work with CsPbCl_3_ and tested with a 6 MV photon beam from a clinical RT linac proved indeed to meet all the above mentioned strict requirements.

## 2 Materials and Methods

Cesium lead chloride (CsPbCl_3_) films have been deposited by Radio Frequency (RF) magnetron sputtering on plastic substrates equipped with an array of Cu electrodes [15], [16], [17]. The mechanochemical procedure described in [18] has been used to obtain the CsPbCl_3_ powder. The procedure consists in the grinding of the two precursor salts (CsCl and PbCl_2_) in equal molar ratio in a mixer mill (Retsch model MM400). The sputtering target is a 5 cm diameter disk realized by pressing the perovskite powder by means of a pneumatic press (11.5 MPa working pressure) for 24 h at 150 °C. The magnetron sputtering equipment is a Korvus HEX (Korvus Technology Ltd.) equipped with an RF source working at 13.56 MHz. The deposition has been performed at room temperature with an RF power of 20W and an argon gas flow of 20 sccm, dynamic working pressure of 2 x 10^−6^ atm and deposition rates in the range 5 - 7 x 10^−2^ nm s^−1^. The film thickness has been monitored during growth by using a quartz crystal microbalance. During the deposition the sample holder was rotating to ensure thickness homogeneity across all the substrate.

**Figure 1:**
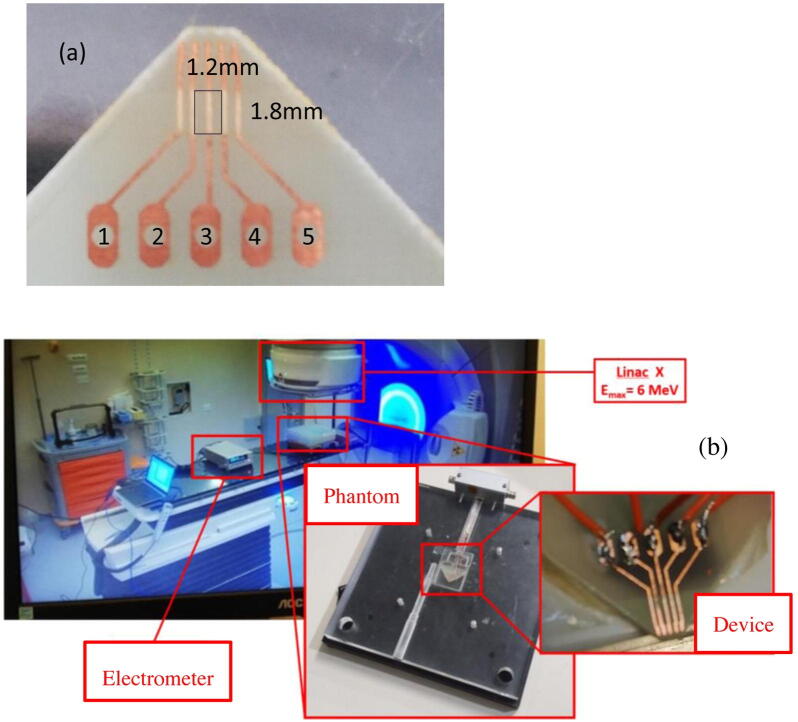
(a) 1.3 μm thick CsPbCl_3_ film deposited by magnetron sputtering on a plastic substrate equipped with a linear array of 5 Cu electrodes, (b) the set-up for dosimetric characterization of the CsPbCl_3_ device at the Radiotherapy Unit of the University of Florence with the device placed in the PMMA phantom.

Fig. 1 (a) shows a CsPbCl_3_ 1.3 μm thick film grown by magnetron sputtering on the plastic substrate equipped with an array of five linear Cu electrodes with 0.6 mm pitch. The five electrodes are numbered in the figure. The active area used in this work, with size 1.2 mm x 1.8 mm, is shown as a black solid line. It corresponds to electrical connections between electrodes 2 and 4. The dosimetric characterization of the device has been performed at the Radiotherapy Unit of the University of Florence. A 6 MV photon beam delivered by an ELEKTA Synergy BM linac has been used. The monitor units delivered by the linac during irradiation are read-out by ionization chamber placed in parallel, the dose is then calculated as 1 Mu = 0.01 Gy. The device has been encapsulated in PolyMethylMethAcrylate (PMMA) to achieve electronic equilibrium during irradiation. Fig. 1(b) shows the film sandwiched in the PMMA phantom and positioned on the linac couch. Measurements have been performed with the detector at a depth of 10 cm and at a 100 cm distance from the linac source. Two electrodes of the CsPbCl_3_ device have been connected via a triax connection to a Keithley 6517 high resistance source/electrometer, used both to supply an external voltage, typically in the range ±50 V and to read-out the current**,** in the range 10 nA - 0.1 pA. The internal circuit of the instrument allows to monitor the current in real-time within 10 ms integration time.

In terms of experimental methodology, we proceeded as follows. We first verified through optical and electrical measurements that the deposited film, CsPbCl_3_, is characterized by an high optical quality and high electrical resistivity, obtained as a combination of the wide band-gap and the high morphological purity. This is necessary to guarantee low dark currents during real-time measurements, as the background currents are main contribution to noise during irradiation. Then, a set of measurements during irradiation with the 6 MV photon beam at same dose-rate was carried out to evaluate in which operational conditions best signal-to-noise ratio are obtained. Crucial issues concerning the dosimetric characterization of our device, as linearity of the collected charge with the absorbed dose and of the current signal with the dose-rate, have been inspected in separated sets of measurements, in ranges relevant for conventional radiotherapy applications. A further set of measurements has been carried out to inspect the electrical structure, either ohmic or diode-like, of our device. This has been performed by irradiating it both under the 6 MV photon beam with different dose-rates at null bias and by measuring the I-V characteristics under irradiation with a constant dose-rate. Through a numerical analysis of these data, an equivalent circuit has been then extracted in the discussion. This latter has been finally used to estimate, through the evaluation of the mean ionization energy of the material, the sensitivity per unit volume of the CsPbCl_3_.

## 3 Experimental results

**Figure 2:**
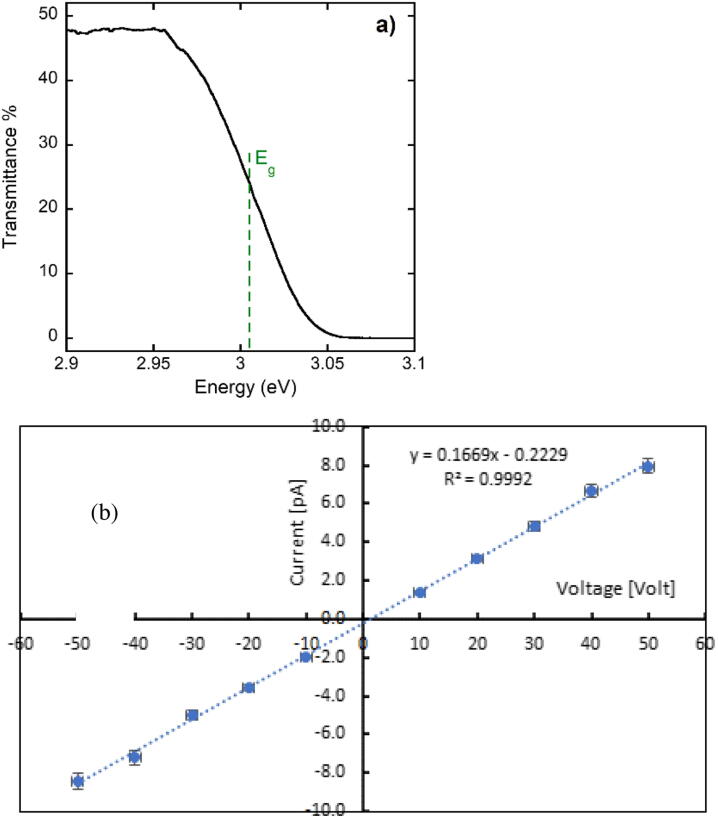
Optical and electrical characterization of the CsPbCl_3_ film grown by magnetron sputtering. (a) Transmittance spectrum at room temperature; (b) Current – voltage characteristics measured at room temperature in dark (electrodes on contact pads 2 and 4).

Fig. 2 (a,b) shows the optical and electrical characterization of the CsPbCl_3_ film. Fig. 2 (a) reports transmittance spectrum measured at room temperature with films of the same thickness grown on glass substrates: bandgap obtained from this measurement is 3.0 eV. Fig. 2 (b) shows the current – voltage characteristics measured in dark with electrodes on contact pads 2 and 4, placed in the PMMA phantom. The function is linear, indicating an ohmic behavior with resistance R_dark_ = 5x10^12^ Ohm. The corresponding resistivity measured at room temperature (23 °C) is ρ = 5 GΩ m. This high value is a consequence of both the wide bandgap of the material and the high morphological quality of the film.

**Figure 3:**
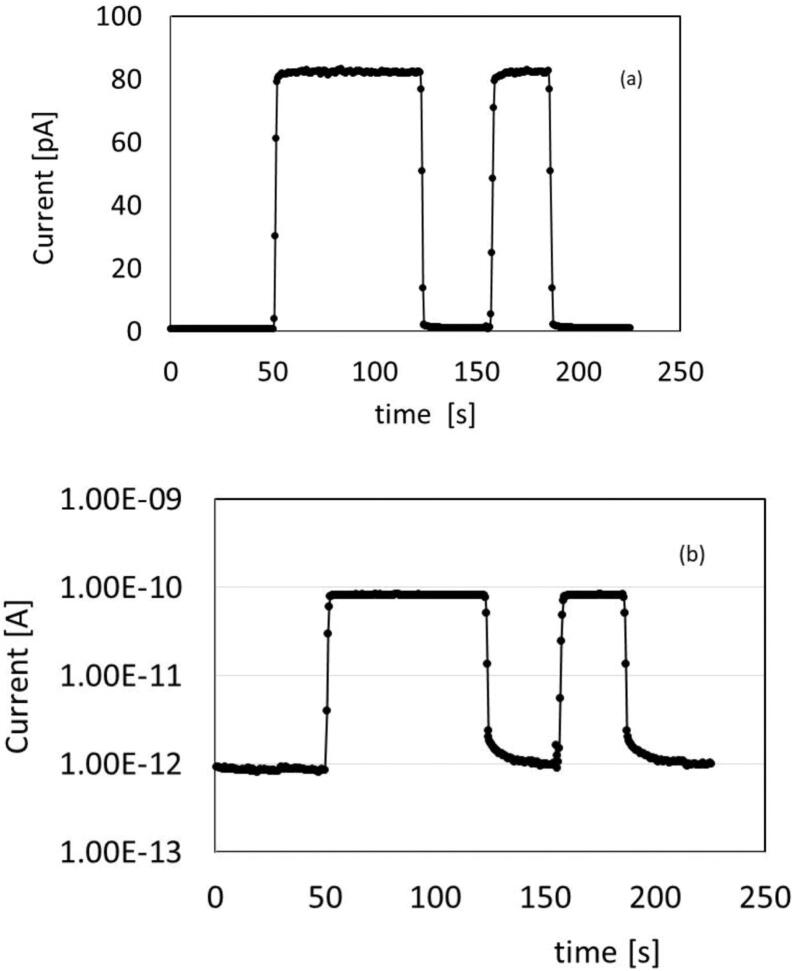
CsPbCl_3_ device measured under a 6 MV photon beam from a linac when a 10 V external voltage is applied between 2 and 4 electrodes. (a) Current signal for two consecutive irradiations with different doses (2 Gy and 1 Gy) but equal dose rate of 4.26 Gy/min. (b) Same data shown in a logarithmic scale to visualize both the signal and the background current measured when the beam is off.

Fig. 3 (a,b) shows the dosimeter signal under the 6 MV photon beam, 10 V applied between electrodes 2 and 4. Shown is the current signal for two consecutive irradiations with different doses (2 Gy and 1 Gy) but equal dose rate of 4.26 Gy/min. Fig. 3 (a) shows the signal in linear scale to evidence fast rise/decay times, stability of the signal, absence of overshoot effects, repeatability of the current signal during two consecutive pulses. The plot in Fig. 3 (b) is in logarithmic scale to visualize both the signal and the dark current measured when the beam is off.

**Figure 4:**
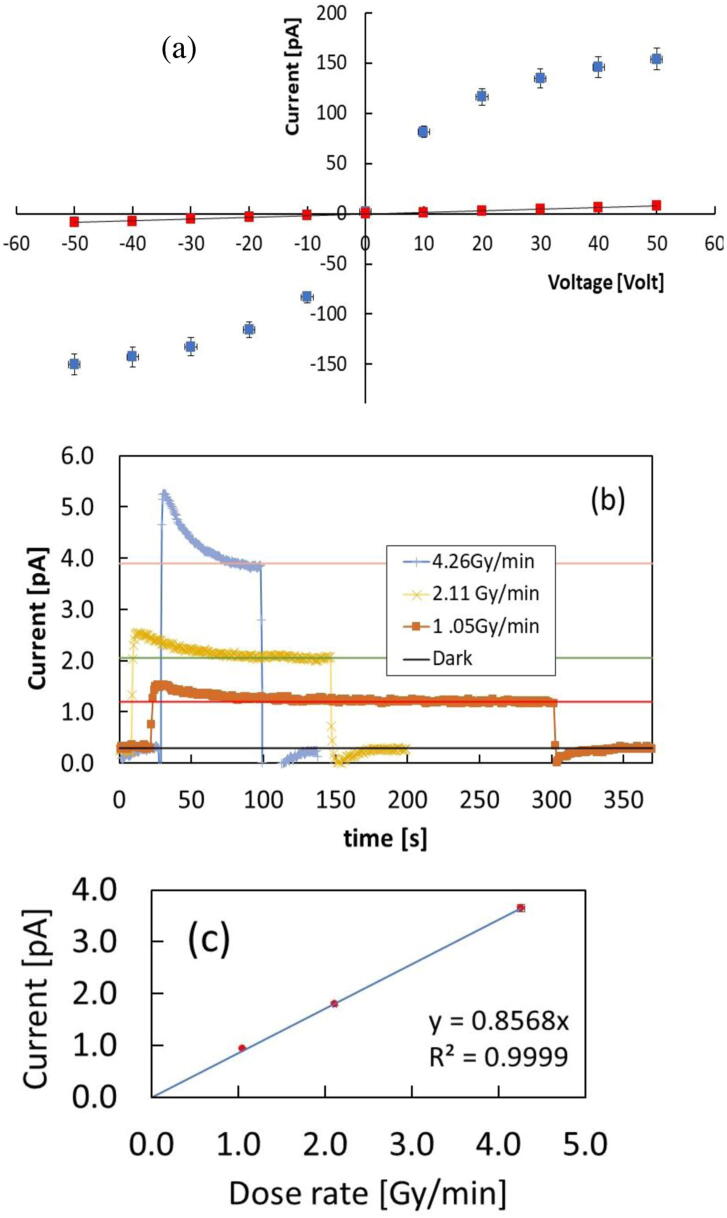
(a) I-V characteristics of the CsPbCl_3_ device measured under a 6 MV photon beam from linac with 4.26 Gy/min dose rate (blue) and in dark (red) (b) Current signal measured with the CsPbCl_3_ device under the 6 MV photon beam with three different dose rates, null bias applied. (c) Current signal as a function of the dose-rate and best-fit showing a linear behavior.

Measurements have been repeated with different external voltages, in the range ±50 V and same dose rate, 4.26 Gy/min. Fig. 4 (a) reports the average current measured at each voltage plotted as a function of the voltage, together with the average background current measured as a function of the voltage when beam is off. We observe that under irradiation the current follows a sublinear, almost symmetrical, function of the voltage. The non-ohmic I-V characteristics under irradiation can be explained considering the formation of Schottky barriers at the electrode/semiconductor interfaces, as it will be discussed in the next section. To test the presence of blocking contacts at interfaces we measured the response of our device under 6 MV photon beam in photovoltaic mode, namely with no bias applied across electrodes. Fig. 4 (b) shows the signal measured at three different dose rates: 1.05 Gy/min, 2.10 Gy/min, 4.26 Gy/min measured at null bias. Since the Elekta accelerator controls the dose rate via the pulse rate, it is important to mention here that the dose per pulse is the same for all irradiations. The current signal plateau achieved at every dose-rate is stable and significantly higher than the dark current (S/N > 4). Moreover, the signal plateau increases linearly with the dose-rate (see Fig. 4 c) while the dark noise is almost the same for the all set of measurements. This may be indeed explained considering the existence of an intrinsic, built-in electric field at the semiconductor/electrode interface, accelerating carriers towards electrodes.

**Figure 5:**
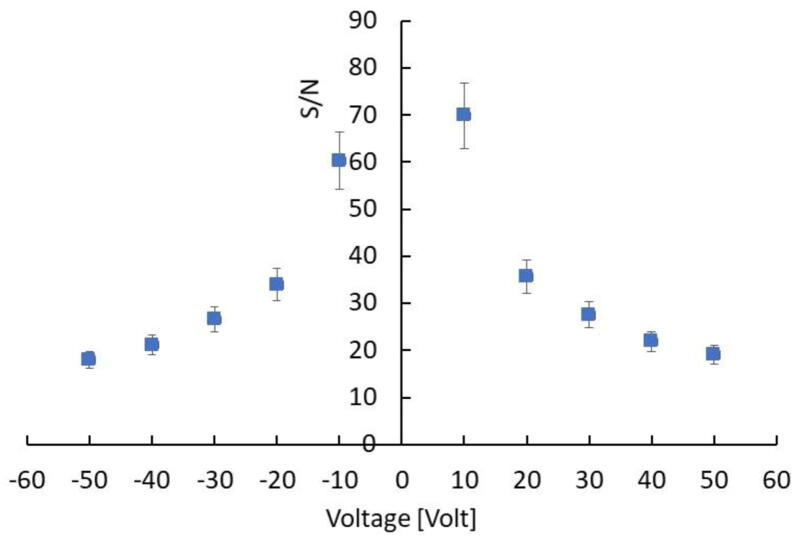
Signal-to-noise ratio measured with the CsPbCl_3_ device under the same 6 MV photon beam and dose-rate as a function of the external voltage.

The plot in Fig. 5 shows the signal-to-noise ratio, S/N, obtained by dividing the average current measured at each voltage during irradiation, with the current measured at the same voltage when the beam is off. The result is a function with highest values, S/N ≈ 60-70, measured at ±10 V: then it decreases and almost flattens at high voltages.

**Figure 6:**
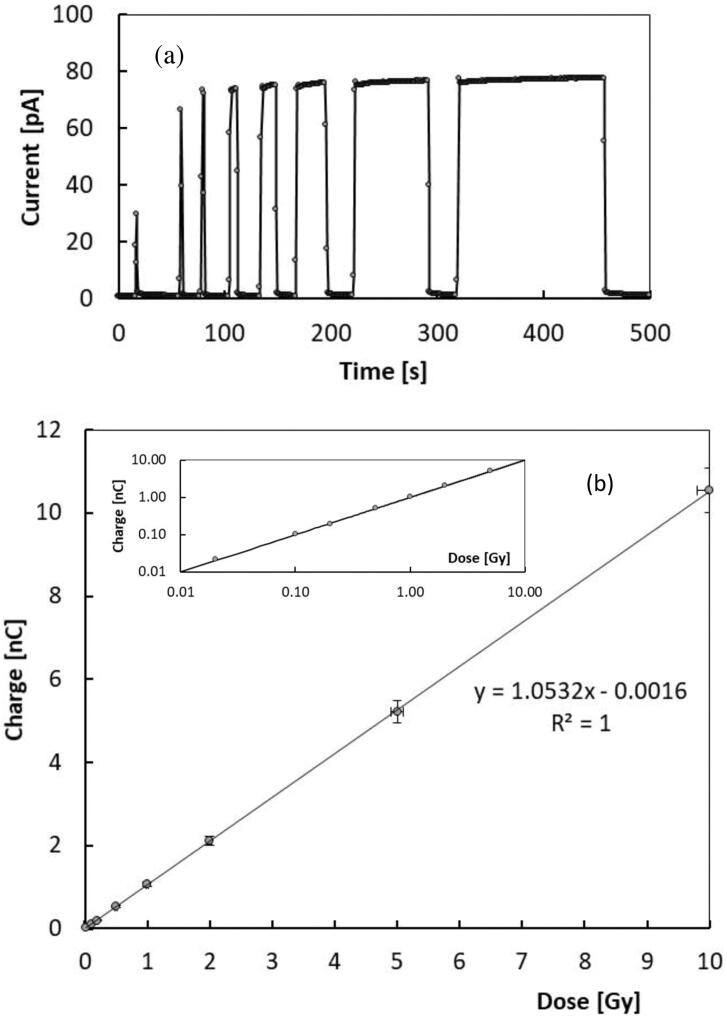
(a) Current signal of the CsPbCl_3_ device with 10 V applied voltage, measured under a 6 MV photon beam with 4.26 Gy/min dose rate, at increasing doses from 0.02 Gy to 10 Gy. (b) Charge collected as a function of the dose, obtained integrating signals of Fig. (a). Log-log plot in inset evidences that linearity holds on over three orders of magnitude.

Fig. 6 (a) shows the current signal measured with the CsPbCl_3_ device (10 V applied between electrodes 2 and 4, highest S/N condition) under the 6 MV photon beam with same dose rate, 4.26 Gy/min at increasing doses: 0.02-0.1-0.20-0.50-1-2-5-10 Gy. The charge collected at each irradiation is plotted as a function of the dose in Fig. 6 (b). The plot evidences a linear trend: log-log plot in inset shows that linearity holds on over three orders of magnitude. The slope of this linear function is the sensitivity of the film: S = 1.05 nC/Gy.

**Figure 7:**
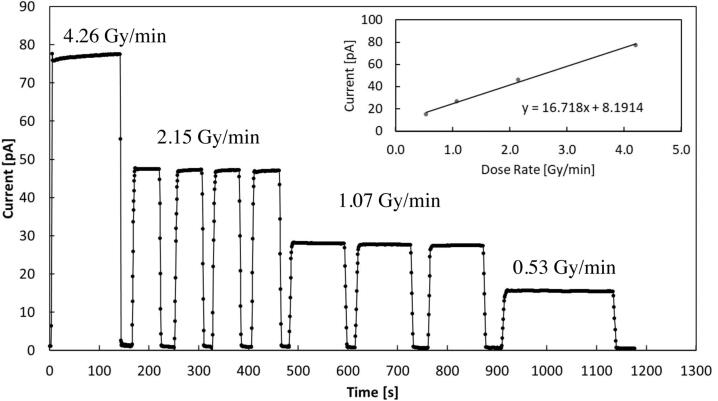
Current signal measured by the CsPbCl_3_ device, 10 V applied voltage under a 6 MV photon beam from linac with different dose-rates. Inset: Mean current at each dose-rate plotted as a function of the dose-rate and linear best-fit.

Fig. 7 shows the current measured by the CsPbCl_3_ device with a 10 V applied voltage, under the 6 MV photon beam from linac with 4 different dose-rates. Inset shows the mean current measured as a function of the dose-rate. The signal is indeed linearly dependent on the dose rate: from the slope we get a sensitivity S = 1.00 nC/Gy, quite in good agreement with results obtained by the linear fit of the charge *vs* dose curve, considering the uncertainty of the measurement, about 5%.

## 4 Discussion

**Figure 8:**
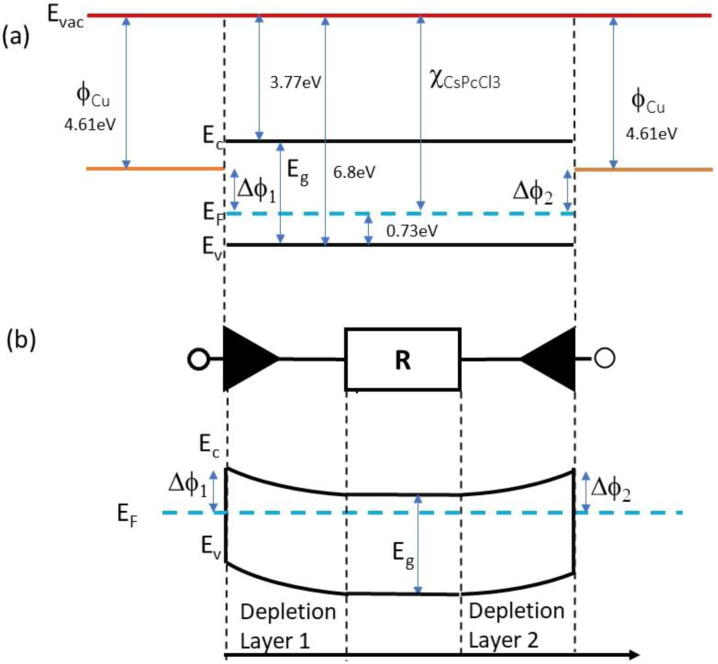
(a) Energy diagram of isolated Cu/CsPbCl_3_/Cu materials in case of p-type conductivity of the semiconductor. (b) Sketch of the equivalent circuit of our Cu/CsPbCl_3_/Cu two-terminal device, made of a resistence in series with two back-to-back Schottky barriers, band diagram evidences the bending due to the Schottky barriers at equilibrium.

Results in Fig. 3 (a) show that, under irradiation, the current signal as a function of the voltage is characterized by a sublinear, almost symmetrical behavior. The non-ohmic I-V characteristics under irradiation can be explained considering the formation of Schottky barriers at the electrode/semiconductor interfaces, as discussed below. Measurements performed at null bias support this hypothesis. The energy diagrams of isolated Cu/CsPbCl_3_/Cu materials are shown in Fig. 8 (a), where we take into consideration the p-type conductivity of the semiconductor and values of conduction band minimum and valence band maximum as given in [19]. The equivalent circuit of our detector is shown in Fig. 8 (b), togeher with the band diagram including band bending at the two Cu/CsPbCl_3_ interfaces, due to the alignment of the Fermi level at equilibrium. It is a two-terminal configuration where the two Schottky barriers at electrode/semiconductor interfaces are connected back-to-back through the resistance R, given by the CsPbCl_3_ homogeneous film in between. Being in series, the current flowing through the three components will be the minimum among that of the three elements alone. When the resistance R of the semiconductor is sufficiently high, as in dark, it will limit the current flowing across the two Schottky diodes and the I-V characteristics will appear as ohmic. Conversely, during irradiation the resistance decreases due to the generation of electron-hole pairs, the I-V characteristics is then limited by that Schottky barrier in reverse condition. Barriers originate due to band bending at interfaces when materials are in contact.

**Figure 9:**
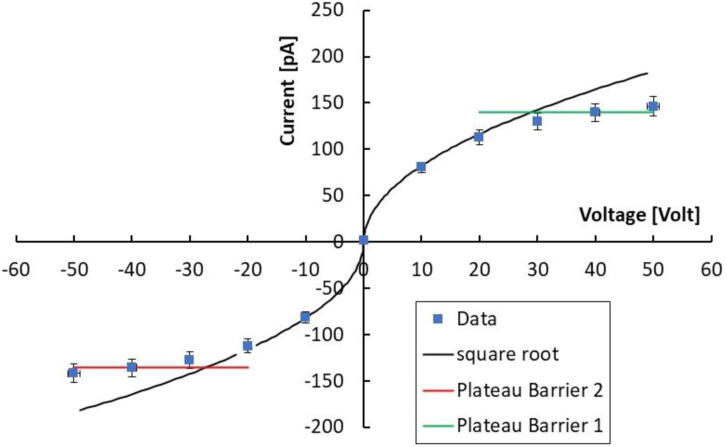
Current -voltage characteristics of the CsPbCl_3_ device measured under 6 MV X-photons with a dose rate 4.26 Gy/min. Best-fit (black solid line) is obtained as a square-root function of the voltage within ± 30V. For higher applied voltages, the I-V characteristics is flattening, green and red lines evidence plateau values.

The current across a metal/semiconductor/metal device characterized by two interfaces both presenting a blocking character can be modeled as in [20]. In case of wide band-gap materials, the resistance of the semiconductor film between the metal contact is usually high, so the potential drop at the resistance cannot be neglected. The I-V characteristics is expressed as:

(1) $$I\left(V\right)=\frac{I_{01}I_{02}sinh\left(q\frac{V-IR}{2KT}\right)}{I_{01}exp\left(q\frac{V-IR}{2KT}\right)+I_{02}exp\left(-q\frac{V-IR}{2KT}\right)}$$

where V = V_1_ + V_2_ is the sum of the potential at the two barriers, I = I_1_ = -I_2_ is the current flowing through the device and $I_{01,02}=AA^{*}T^{2}\text{exp}\left(\frac{q\varphi_{1,2}}{KT}\right)$ with A device section, A* Richardson constant, and $\varphi_{1,2}$ the Schottky barrier heights at the two contacts. A first simulation of the I-V curve in these ideal conditions can be obtained considering $\varphi_{1,2}\approx 0.5V$; R ≈ 6x10^10^ Ω. Nonetheless, in a real diode produced with a wide-bandgap material kept in reverse condition, generation currents within the depleted region must also be considered. Due to Shockley-Read-Hall (SRH) mechanisms [21] generation currents arise which depend linearly on the depletion thickness, which in turn is proportional to the square root of the applied reverse bias. Thus: $I\propto \sqrt{V}$. This square-root dependence must be observed only until the device is partially depleted, namely until the depletion depth has achieved the total thickness of the device. In this case, ideally, the reverse current flattens to a constant value. Practically, by further increasing the voltage, a smoother increase of the current is observed due to surface and perimetral current contributions, if guard-rings are not present. Fig. 9 shows the best-fit of the current as a function of the voltage, measured under the 6 MV photon beam with a dose rate 4.26 Gy/min, considering the SRH model. Best-fitting with a square-root function of the voltage is actually very accurate in the range within ± 30V. For higher voltages the current data are flattening: only a slight increase of current is observed increasing further the voltage, suggesting that full depletion is indeed achieved at V_dep_ ≈ 30 V.

We may then evaluate the sensitivity per unit volume of the material in full-depletion conditions. The plateau value of the current measured at high voltages, when we suppose the full depletion is achieved, is: $I_{dep}=138\;\text{pA}$. Considering the corresponding dose-rate of D_r_ = 4.26 Gy/min, we get: $S_{dep}=I_{dep}/D_{r}=1.94{\text{ nC Gy}}^{\text{−1}}$, almost twice the sensitivity measured at 10 V. Considering the plateau signal as due to the charge collected from the total volume of the device, 3x10^-3^ mm^3^, we determine the sensitivity per unit volume: $S_{V}=640\frac{nC}{{\text{Gy mm}}^{3}}$. It is then possible to write the sensitivity per unit volume as [6]: $S_{V}=S/Volume$ $=qG/D_{r}$, with q electronic charge and $G=D_{r}\rho /E_{i}$ e-h pair generation rate. Considering ρ mass density and E_i_ mean ionization energy we finally get: $S_{V}=q\rho /E_{i}$.

Considering the sensitivity per unit volume value measured in this work and the mass density, ρ = 4.22 g/cm^3^ [22], we get $E_{i}=6.6\;\text{eV}$ for magnetron sputtered CsPbCl_3_ films. To briefly discuss this result, we observe that for many conventional semiconductors, as silicon and diamond, E_i_ can be expressed as a linear function of the bandgap, E_g_, through the semi-empirical Klein's law [23]:

(2) $$E_{i}=\frac{14}{5}E_{g}+r\left({\hbar \omega }_{r}\right)$$

**Figure 10:**
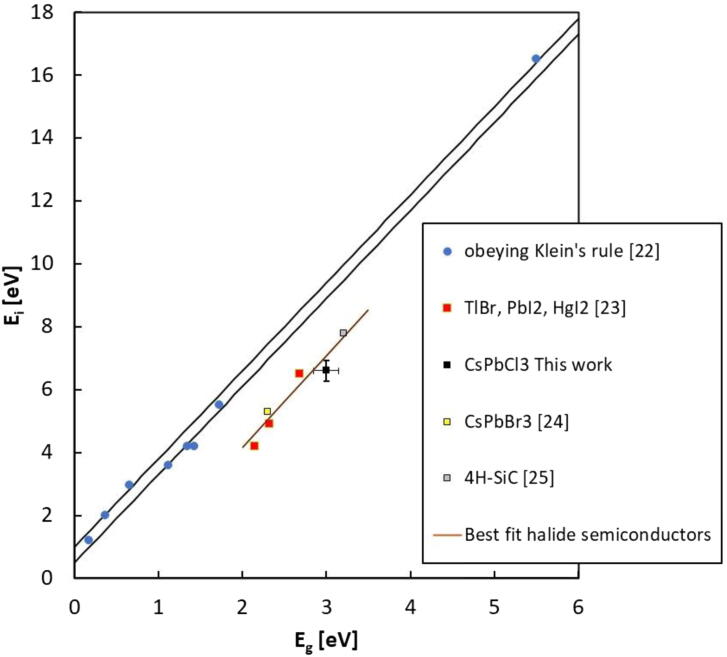
Mean ionization energy of various semiconductors plotted as a function of the energy gap. Region within the two black lines indicates the range covered by the Klein's rule in eq. (2). Those semiconductors obeying the Klein's rule are shown in blue. Other data concern materials not obeying the rule. Linear best-fit for halide semiconductors is also given as a guide-line. Mean ionization energy measured in this work for magnetron sputtered CsPbCl_3_ film is shown as a black square, in green the value for crystalline CsPbCl_3_ samples [25].

Here, $E_{i}$ is calculated as a sum of three contributions: the bandgap, E_g_, the optical phonon losses, r(ħω_r_) usually in the range 0.5 eV - 1.0 eV and the residual kinetic energy, estimated as 9/5 E_g_. Nonetheless, halide semiconductors such as HgI_2_, TlBr, PbI_2_ [24], and more recently the metal halide perovskite CsPbBr_3_ [24], appear to be characterized by mean-ionization energies E_i_ smaller than those predicted by eq. (2). Our result evidences that also CsPbCl_3_ should belong to this class of materials. In fact its mean-ionization energy, as determined by the Klein's rule, should lie in the range: 8.8-9.4 eV, much higher than what measured in this work. Fig. 10 reports (E_g_ *,* E_i_) pairs of several semiconductor materials, together with guide-lines for both material classes: either obeying the Klein's rule or not. The value obtained in this work for CsPbCl_3_ is within the guide-line tracing the behavior of halide semiconductors non respecting the Klein's rule.

Table 1 summarizes main parameters for silicon, SiC, diamond, materials already of interest for high-energy radiation detection, with results obtained for metal halide perovskites: CsPbBr_3_ in [12], [25] and CsPbCl_3_ in this paper.

**Table 1:** Parameters of CsPbCl_3_ thin films obtained in this work as compared with those given in literature for semiconductor materials relevant in high energy radiation monitoring.

	Silicon	SiC	Diamond	CsPbBr_3_	CsPbCl_3_
ρ (g/cm^3^)	2.33	3.2	3.5	4.5	4.2
E_g_ (eV)	1.12	3.3	5.5	2.3	3.0
E_i_ (eV)	3.60	7.8	13	5.3	6.6
S_V_ (nC/Gy mm^3^)	637	411	217	860	640
Refs.	[6]	[26]	[8]	[12]	This work

## 5 Conclusions

Magnetron sputtered CsPbCl_3_ thin films grown on plastic substrates equipped with Cu electrode arrays have been investigated for the first time as dosimeters in clinical radiotherapy. Our results prove that CsPbCl_3_ belongs to the class of halide semiconductors, characterized by a value of the mean ionization energy for high – energy radiation definitely lower than that of conventional semiconductors with same energy bandgap used for radiation detection. Indeed, our prototype devices appear very promising, coupling together a high sensitivity per unit volume, comparable to that of silicon, the most sensitive material used for dosimeters, with very low dark current at room temperature, due to the significantly higher bandgap of CsPbCl_3_ against that of silicon. For these reasons, a high signal-to-noise, up to 70, has been measured under a 6 MV RX beam at moderately low applied voltages in real-time. Linearity of the sensitivity with the dose has been verified over three order of magnitudes, linearity of the current signal with the dose rate has been also successfully tested in the range 0.5-4.3 Gy/min. Schottky barrier back-to-back structure at material/electrode interfaces, allows to confine the active volume of the dosimeter on submillimetric sizes. This is a very important feature with respect to the manufacturing of monolithic two-dimensional detector arrays, which are characterized by high granularity and high spatial resolution. Moreover, the blocking contact allows for operating in null applied voltage conditions, quite appealing for in-vivo applications. In turn, the possibility to finely control the thickness of the film during the sputtering process, by a proper selection of the deposition-rate allows for growing thin active layers on almost any kind of substrates, to manufacture optimized devices for transmission measurements. Our study proves indeed that metal halide perovskite CsPbCl_3_ may open the way to a novel generation of flexible, high spatial resolution, thin transmission detectors for in-vivo and real-time applications in RT. Our research program on perovskite radiation detectors will thus continue in near future, with the goal of investigate realistic applications of such a promising materials for medical physics. To definitely assess their use as clinical dosimeters it will be of great importance to study the long-term effects of the high total doses accumulated over time. It would be equally important to find out how the proposed detectors perform at extremely high dose rates, as this represents a future new area of application in radiotherapy. Further, the development of clinically applicable in vivo detectors would be of great interest. In this respect, future studies will concern the deposition of CsPbCl_3_ on flexible interdigitated electrodes adapting to the complex geometry of human body and in transmission mode. Other important dosimetric characterization, as energy dependence and dose per pulse dependence will be also the subject of forthcoming works in view to finalize a dosimetric device based on inorganic CsPbCl_3_ perovskite.

## Declaration of Competing Interest

The authors declare that they have no known competing financial interests or personal relationships that could have appeared to influence the work reported in this paper.
